# Progress of Medicine

**Published:** 1882-05

**Authors:** 


### PROGRESS OF MEDICINE.

Variola.—Dr. J. T. Marcley, of Buffalo, wrote notes of twenty-one cases of small-pox, and thus summarizes some of their interesting and instructive features:

First, in regard to the occurrence of the different symptoms.

Chills were present in all cases, in some quite severe.

Headache of a more or less violent character was present in all.

Backache occurred in ten, but was violent in only one (hæmorrhagic).

Nausea was present in all; vomiting, in eleven; violent and long-continued in only one case.

The tongue was much coated in all cases, but was not the seat of much eruption, only in two.

Sore throat was complained of in eight, severe in only two.

Delirium was present in four, very violent only in the hæmorrhagic case.

Abdominal pain in one case.

Constipation was marked in all cases; convulsions occurred in only one case.

Itching was distressing in two or three only.

Conjunctivitis was present in four cases, severe in only one.

Pain in hands and feet was complained of in six cases, severe only in the hæmorrhagic.

Œdema of lower extremities was marked in only one case.

Loss of hair occurred only in one, to any extent.

Pulse, owing to the difficulty in getting at this symptom, on account of swelling, it was neglected.

In regard to the period at which the eruption appeared in those cases that I could follow closely, it varied from two to five days from the beginning of the initial fever.

The period of incubation was twelve days in seven cases, and in three, fourteen days.

In regard to vaccination, thirteen had been vaccinated, none, however, within a number of years. Eight had never been vaccinated. Of those vaccinated, the disease assumed the form of varioloid in five cases. Distinct in one, coherent in four, confluent in two, and hæmorrhagic in one case. Of those not vaccinated, distinct in one, confluent in six. One died before the eruption appeared. In only one case was there any history of a successful (or non-successful) re-vaccination. This one had the disease the lightest of any, only a dozen spots appearing.

Although these cases are few in number, they speak very strongly in favor of vaccination, and especially re-vaccination.

The treatment of these patients was very simple. Anodynes and cathartics, or rather, laxatives and stimulants, as required. Potass. chlorat. was found very good in the sore throat of this disease. Locally, I found nothing so agreeable to the patient as olive oil, and as far as preventing the pitting goes, I do not see but it proves as satisfactory as anything else.

The method followed in disinfection, was the burning of sulphur in tightly closed rooms, leaving them so for several hours, then thoroughly airing them; washing the floors, walls, ceilings, wood-work, and all wooden articles of furniture with a strong solution of carbolic acid, and by burning all cloth goods (bedding, curtains, carpets, etc.) that were in the least infected.—*Buffalo Medical aud Surgical Journal.*

